# Mechanism of the feedback-inhibition resistance in aspartate kinase of *Corynebacterium pekinense*: from experiment to MD simulations†

**DOI:** 10.1039/d0ra09153g

**Published:** 2020-12-22

**Authors:** Xiaoting Liu, Caijing Han, Li Fang, Zhanqing Fan, Yanan Wang, Xin Gao, Junhua Shi, Weihong Min

**Affiliations:** College of Food Science and Engineering, National Engineering Laboratory of Wheat and Corn Deep Processing, Jilin Agricultural University Changchun 130118 Jilin People's Republic of China minwh2000@jlau.edu.cn +86-431-8451-7235 +86-139-4491-9697; National Engineering Laboratory of Wheat and Corn Deep Processing Changchun 130118 Jilin China; School of Public Health, Weifang Medical University Weifang 261042 Shandong China

## Abstract

In microorganisms and plants, aspartate kinase (AK) is the initial committed enzyme of the biosynthesis of the aspartate acid family amino acids and is inhibited by end products. In the paper, we mutated the key allosteric regulatory site A380 around the binding site of the Lys inhibitor in *Corynebacterium pekinense* AK (*Cp*AK). A single-mutant A380C was obtained with 12.35-fold higher enzyme activity through high-throughput screening. On this basis, T379 as another key allosteric regulatory site was further modified, and the double-mutant T379N/A380C with 22.79-fold higher enzyme activity was obtained. Molecular dynamics (MD) simulations were used to investigate the mechanism of allosteric inhibition by Lys. The results indicated that the binding of Lys with *Cp*AK resulted in conformational changes and a larger distance between the phosphorus atom of ATP and the oxygen atom of Asp, which was detrimental for the catalytic reaction. However, the mutation of allosteric sites opens the “switch” of allosteric regulation and can prevent the conformational transformation. Some key residues such as G168, R203, and D193 play an important role in maintaining the substrate binding with *Cp*AK and further enhance the enzyme activity.

## Abstract 2

*Corynebacterium pekinense* AK was successfully modified and two mutants A380C and T379N/A380C with high enzyme activity were constructed. The mechanism of their feedback-inhibition resistance was thoroughly studied from experiment to MD simulations.

## Graphical abstract

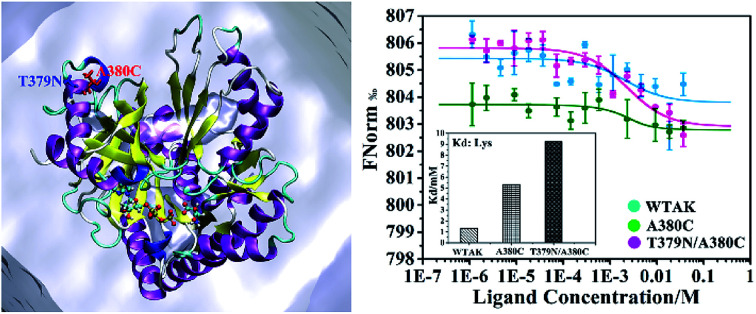

## 1. Introduction

Aspartate kinase (AK, EC 2.7.2.4), also named aspartokinase, is the first and typical allosteric enzyme responsible for the synthesis of the aspartate acid family amino acids including lysine, threonine, methionine, and isoleucine.^1^ AK catalyzes phosphorylation of the γ-carboxylate group of aspartate in microorganisms and plants and is regulated by end-products through feedback inhibition.^2^ For instance, AKIII from *Escherichia coli* and *Ca*AK from *Clostridium acetobutylicum* are sensitive to Lys,^3,4^ while *Mj*AK and *Tt*AK separately found in *Methanococcus jannaschii* and *Thermus thermophilus* are both subjected to the feedback inhibition by threonine.^5,6^ Additionally, AKI from *Arabidopsis thaliana* is inhibited synergistically by lysine and *S*-adenosylmethionine,^7^ whereas *Cg*AK (from *Corynebacterium glutamicum*) and *Sy*AK (from *Synechocystis*) are sensitive to Lys plus Thr in a concerted manner.^8,9^

So far, most structurally determined AKs are homo-oligomeric enzymes composed of α subunits, while *Cg*AK, *Mj*AK, *Tt*AK, and *Pa*AK (from *Pseudomonas aeruginosa*) display a unique α_2_β_2_-type heterotetrameric structure which contains equimolar α and β subunits encoded by in-frame overlapping genes. In this α_2_β_2_-type AK, the N-terminal portion of the α subunit serves as a catalytic domain, whereas the β subunit and the C-terminal region of the α subunit function as regulatory domains. The catalytic domain is further divided into two lobes, the N-lobe making up the Asp-binding site and the C-lobe offering a nucleotide-binding pocket for ATP. A key feature of the regulatory domain is the conserved ACT domain which is found in a wide variety of allosteric enzymes.^10^ In recent years, the allosteric regulation of AK has attracted profound attention from scholars. The general allosteric mechanisms of AK have been concluded: the effector binding leads to a conformational transition from a substrate-ATP bound relaxed state (R-state, close conformation) to the inactive tense state (T-state, open conformation), which in turn hinders the catalytic reaction.^3,11^

To promote the industrial production of the Asp-derived amino acids, engineering of regulation and activity of AK is important. Therefore, feedback-resistant mutations are successfully made in genes encoding for LysC (aspartate kinase).^2,9,11^ Apart from the enzymological interest, AK is an attractive target for developing new antibiotics and antitubercular drugs because of its absence in human and animals.^12^ For example, *Pa*AK plays crucial roles in biofilm-associated antibiotic-resistance and infections, which demonstrates its potential application in anti-*P. aeruginosa* treatment.^13,14^*Mycobacterium tuberculosis* (*Mtb*) AK has been identified as a potential drug target in *Mtb*, especially in multidrug-resistant *Mtb*.^12,15^

**Fig. 1:**
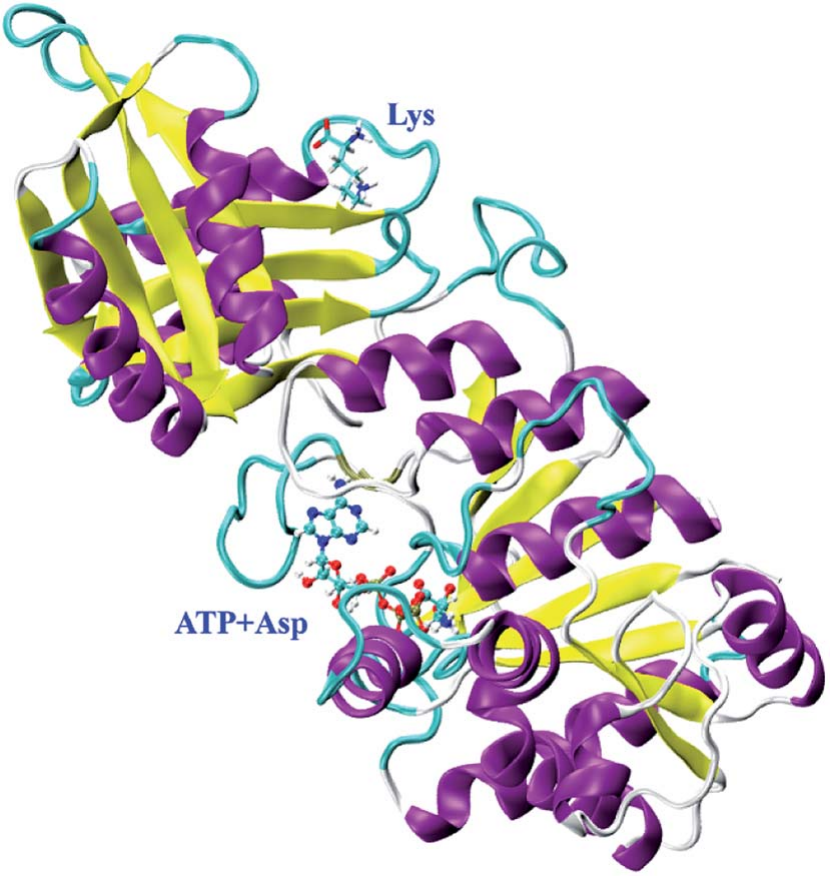
Structure of *Cp*AK.

In our previous study, *Cp*AK from *Corynebacterium pekinense* has been found to be a novel allozyme (Fig. 1) and T379 as well as A380 around the binding site of inhibitor Lys are the conserved allosteric regulatory sites. Elucidation of its allosteric regulatory mechanism is of great significance for the construction of highly efficient microbial strains.^16–18^ However, up to date, the detailed allosteric mechanism of *Cp*AK is still unclear. In this work, we designed and expressed novel mutants with high-enzyme activity as well as feedback inhibition resistance on the basis of sites 379 and 380 by using site-directed mutagenesis and high-throughput screening techniques. Furthermore, molecular dynamics (MD) simulations and microscale thermophoresis (MST) were applied to study the interaction between AK and substrate or inhibitor, which sheds light on the allosteric regulation.

## 2. Results and discussions

### 2.1. Construction and purification of mutant strains

**Fig. 2:**
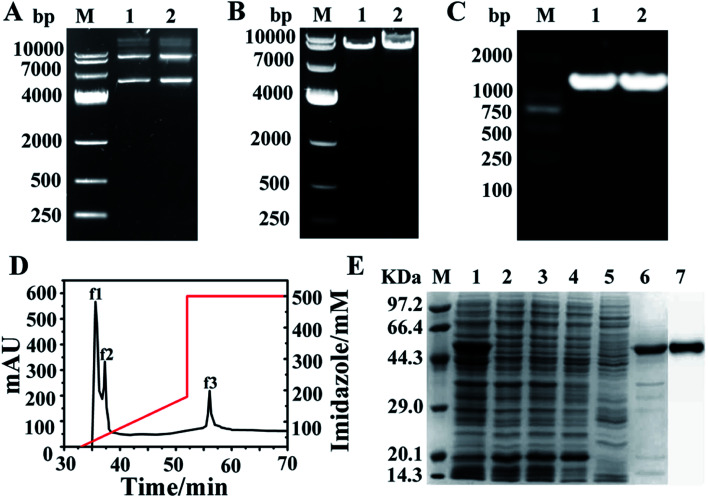
Electrophoretic verification and purification of AK. (A) Agarose gel electrophoresis of plasmid PET-28a–AK. M: DNA marker; 1: A380C; 2: T379N/A380C. (B) Agarose gel electrophoresis of mutation PCR. M: DNA marker. 1: A380C; 2: T379N/A380C. (C) Agarose gel electrophoresis of bacterial PCR. M: DNA marker. 1: A380C; 2: T379N/A380C. (D) Purification of AK by ÄKTA. f1, f2: crude enzyme sample after flowing the His Trap; f3: purified enzyme sample. (E) SDS-PAGE and western blot. M: protein marker; 1: crude enzyme sample; 2–3: f1; 4–5: f2; 6: f3; 7: western blot.

The single mutant A380C and double variant T379N/A380C were obtained by site-directed mutagenesis and high-throughput screening. The recombinant plasmid with about 7000 bp (pET-28a was 5369 bp and the target gene AK was 1266 bp) was used as template to perform PCR (polymerase chain reaction) amplification under the action of mutant primers (Fig. 2A). The target band was also detected at 7000 bp (Fig. 2B), which demonstrated the mutation was successfully induced. AK gene with 1266 bp band was amplified by PCR, applying the mutant strain as the template and the cloning primers as the primers, and then sent to Shanghai Sangong for sequencing (Fig. 2C). The components of the crude enzyme were collected after the purification process, of which the peaks f1 and f2 in Fig. 2D were corresponding with the 2, 3 and 4, 5 bands in Fig. 2E, respectively. There was no obvious band at 48 kDa, implying that the target protein was fully purified by the His Trap column. The stripe 6 (Fig. 2E) corresponding to the peak f3 (Fig. 2D) had an obvious band at 48 kDa, indicating that AK has been purified successfully. The stripe 7 was the results of western blotting, indicating that the purified AK protein was successful.

### 2.2. Kinetics and enzymatic properties

**Fig. 3:**
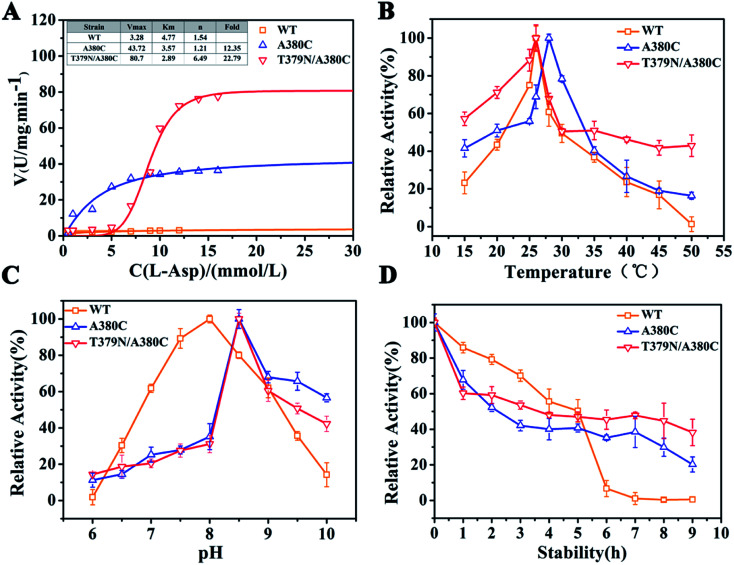
Dynamic analysis and enzymatic properties. (A) Dynamic analysis. (B) The optimum temperature. (C) The optimum pH. (D) The thermal stability.

The kinetic analysis and enzymatic properties of WT (wild-type *Cp*AK), A380C, and T379N/A380C mutant strains were shown in Fig. 3. As shown in Fig. 3A, the reaction rate of WT, single mutant A380C, and double mutant T379N/A380C was 3.28, 40.51, and 80.7 U mg^−1^ min^−1^, respectively. Compared to WT, the enzyme activities of the mutants were increased by 12.35-fold and 22.79-fold, respectively. The *K*_m_ values of WT, A380C, and T379N/A380C were 4.17, 3.57, and 2.89 mM, respectively. The *K*_m_ value decreased gradually, indicating that the enzyme–substrate affinity was enhanced. The *n* value of WT was 1.54, which was a typical allosteric enzyme, manifesting positively synergistic. The *n* values of A380C and T379N/A380C were 1.21 and 1.46, respectively. The smaller is the *n* value, the stronger is the resistance to feedback inhibition.

The optimal temperature of WT, A380C, and T379N/A380C was 26, 28, and 26 °C, respectively (Fig. 3B). The optimal temperature of the A380C variant has risen. Although it has not changed for the double mutant T379N/A380C, its thermal stability increased and retained about 50% of the enzyme activity at 50 °C, which was beneficial to the late fermentation production.^9^ The optimal pH of WT, A380C, and T379N/A380C was 8.0, 8.5, 8.5, respectively, demonstrating that the optimal pH was not improved after mutation (Fig. 3C).

The half-life values of A380C and T379N/A380C mutant strains were 2.22 and 3.27 h, respectively, which were both lower than that of WT (4.5 h), suggesting the relative enzymatic activity values were reduced in Fig. 3D. However, the relative enzyme activity of the mutant strains was higher than that of WT after 6 h, and especially T379N/A380C still maintained above 40% activity after 8 h, implying that the mutations could enhance the thermostability on the whole.

As reported, WT was inhibited by the synergistic feedback of threonine and lysine in a dose-dependent manner.^16–19^ As can be seen from Table 1, the enzyme activity of mutant A380C was improved under different concentration of inhibitors, and even activated at high concentration of Lys, Lys + Thr, Lys + Met, Thr + Met, and Lys + Thr + Met, which was consistent with the decrease of the *n* value in the kinetic analysis. Compared with WT,^16^ mutants had a more obvious disinhibition effect, and the activation effect of mutant is mainly contributed by Lys inhibitor. The activation effect was very clear with the concentration of Lys at 10 mM, indicating that the modified site 380 can effectively remove the inhibition effect. This result lays the foundation for our subsequent research on the allosteric regulation of Lys.

**Table 1:** Effects of different concentrations and inhibitors on A380C and T379N/A380C^a^

Enzyme	A380C				T379N/A380C			
Inhibitors	Relative activity (%)				Concentration (mM)			
	0.2	1	5	10	0.2	1	5	10
Control	100	100	100	100	100	100	100	100
Lys	94.80 ± 0.97	95.74 ± 1.43	99.70 ± 0.54	**103.54 ± 1.32**	96.99 ± 0.56	98.78 ± 4.95	**106.87 ± 0.56**	**115.84 ± 1.81**
Thr	78.26 ± 1.19	80.94 ± 3.06	82.14 ± 3.56	87.80 ± 3.53	94.59 ± 3.26	97.06 ± 0.68	97.84 ± 1.95	98.12 ± 0.44
Met	95.06 ± 1.69	97.71 ± 0.85	98.30 ± 2.6	98.13 ± 1.89	90.47 ± 0.12	96.33 ± 0.56	98.12 ± 0.44	99.47 ± 0.53
Lys + Thr	98.58 ± 2.96	99.09 ± 3.13	**105.48 ± 3.17**	**109.82 ± 3.35**	92.02 ± 0.44	97.44 ± 0.96	**103.57 ± 1.12**	**105.48 ± 0.97**
Lys + Met	97.88 ± 1.47	98.56 ± 2.34	**105.36 ± 2.45**	99.12 ± 4.34	89.61 ± 1.35	93.29 ± 2.65	97.74 ± 3.45	**114.35 ± 1.96**
Thr + Met	97.31 ± 0.86	98.00 ± 1.78	**113.70 ± 3.35**	98.19 ± 3.98	81.87 ± 2.02	90.79 ± 0.75	**104.07 ± 2.53**	92.16 ± 0.65
Lys + Thr + Met	74.04 ± 4.66	80.12 ± 1.03	86.26 ± 1.07	**102.77 ± 3.81**	86.97 ± 1.85	99.21 ± 1.91	**110.01 ± 1.24**	**111.92 ± 2.57**

a The bold indicates activation.

As observed from Table 1, the resistant feedback-inhibition of T379N/A380C was significantly improved with respect to A380C, among which the activation effect was obvious under the action of 5–10 mM Lys, Lys + Thr, and Lys + Met, especially under the action of 10 mM Lys, indicating that the activation effect was regulated by Lys and weakened after mutation. The double mutation may further affect the allosteric regulation of Lys.

The affinity between *Cp*AK and the ligand was further analyzed by MST. As shown in Table 2, the *K*_d_ values of T379N/A380C, A380C, and WT with inhibitor Lys were 9.24, 5.32, and 1.32 mM, respectively. After mutation, the increased *K*_d_ values between inhibitor Lys and variant revealed the lower affinity with inhibitor Lys. The double mutant T379N/A380C had the lowest affinity with the inhibitor Lys, so the disinhibition was relatively obvious. The *K*_d_ values between WT, A380C, and T379N/A380C with substrate Asp (named WT + Asp, A380C + Asp, T379N/A380C + Asp) were 655.08, 313.32, and 309.7 mM, respectively. The decreased *K*_d_ values indicated the binding of AK to the substrate Asp was increased. The *K*_d_ values between WT, A380C, and T379N/A380C and Lys + Asp (named WT + Lys + Asp, A380C + Lys + Asp, T379N/A380C + Lys + Asp) were 701.5, 447.69, and 99.64 mM, respectively. The decreased *K*_d_ values demonstrate strong affinity and high maximum reaction rate. The inhibition of AK was improved after mutation, which was consistent with the inhibitor data (shown in Table 1). The *K*_d_ values of WT + Asp was less than that of WT + Lys + Asp, manifesting its greater affinity, which further identified that Lys played an important role in the allosteric regulation. However, the *K*_d_ value of the mutant strain was significantly lower than that of WT, indicating that the modification around the inhibitor Lys site could eliminate the feedback-inhibition and well regulate substrate binding through allosteric regulation. In the following discussions, molecular dynamics simulation was used for in-depth illustration.

**Table 2:** The *K*_d_ value of AK by MST

Enzymes	*K*_d_ (mM)		
	Lys	Asp	Lys + Asp
WT	1.32 ± 0.00	655.08 ± 0.90	701.25 ± 1.24
A380C	5.32 ± 0.01	313.32 ± 0.79	447.69 ± 0.55
T379N/A380C	9.24 ± 0.00	309.70 ± 0.23	99.64 ± 0.07

### 2.3. Computational analysis

#### 2.3.1. Catalysis analysis on the basis of distance

Each system was subjected to a 120 ns MD simulation to obtain an equilibrated conformation. The structural stability was assessed by the RMSD value of the Cα atoms. To some extent, whether the catalytic reaction can occur or not can be simply judged by the distance between the phosphorus atom of the transferred phosphate (ATP) and the acceptor oxygen atom of the substrate Asp (*d*_Pi–O_).^19–21^ In this work, ATP, as a reactant, is directly involved in the catalysis of the AK enzyme, in which the gamma phosphate group transfers to the carboxyl group of the substrate Asp. The related reaction equation was shown in ref. 16. The calculated *d*_Pi–O_ changes with the simulation time were depicted in Fig. S1 (ESI†). As shown in Fig. S1,† the analysis time of all the simulation systems was 120 ns, in which the first 20 ns was the system equilibrium stage, and the last 100 ns was the production stage for data analysis. All *d*_Pi–O_ values in the 120 ns simulations were given here in order to fully present the changes relative to the starting points in the whole process. Except for the WT + Lys system, the values of *d*_Pi–O_ in the other three systems does not fluctuate significantly. The averages of all the three distances has very small standard deviations, such as 2.78 ± 0.04 Å for the wild-type enzyme, 2.95 ± 0.06 Å for a single mutant A380C, and 2.77 ± 0.04 Å for the double mutant T379N/A380C. However, the *d*_Pi–O_ curve for WT + Lys system fluctuated drastically (4.01 ± 0.60 Å) and ranged from 2.7 to 5.8 Å across an interval of about 3 Å, resulting in noncatalytic pose which would not facilitate phosphoryl transfer.^19–21^ In contrast, the catalytic distances in two mutants were suitable for the reaction. Therefore, combined with the experimental measurements and the MD simulation results, the mutations can resist the feedback inhibition and thus promote the phosphorylation. The detailed inhibitor mechanism of Lys, as well as the feedback inhibition-resistant mechanism of two mutants were elucidated below mainly on ATP and Asp aspects.

#### 2.3.2. Effect on ATP binding

The binding of Lys with WT (named WT + Asp + ATP + Lys system) resulted in a deviation of the allosteric regulatory αA and also affected the shift of αB due to the interaction between these two helices (Fig. S2(A)†). It was supposed that the deviations of αA and αB helices can pass through a remote transmission of interaction and finally caused the whole movement of ATP binding region, especially the tC domain. The shifts would lead to the conformation movement of ATP in comparison with that of WT, which made ATP phosphate far away from the catalytic substrate Asp (Fig. S1†). The increased distance between the reactants was extremely detrimental for the catalytic activity of AK.^21^ Superimposing the structures in the absence and presence of inhibitor lysine indicated that the conformations of the residues around the ATP binding site had no significant change (Fig. S2(B)†). In addition, the calculated heavy atom RMSD value for the residues of the ATP binding site in the two systems is only 1.2 Å, which showed that the relative motion of the above conformation was conceived as a whole and did not destroy the local structure of the ATP binding site, and had little effect on the microscopic interaction between ATP and its related residues.

#### 2.3.3. Effect on Asp binding

**Fig. 4:**
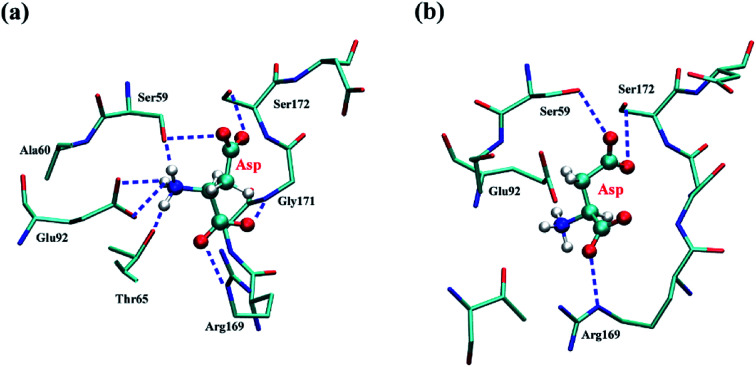
Detailed interaction between the substrate Asp and AK residues. (a) WT system. (b) WT + Lys system.

In addition, the binding of Lys to WT affected the interaction between the substrate Asp and AK. As shown in Fig. S3,† the presence of inhibitor lysine induced a certain deviation of the tA regulatory, which led to a larger movement of the whole dB domain and then the largest movement of AK occurred in the tB region. The change of the calculated RMSD values of the residues in the Asp binding site before and after inhibitor lysine binding is 3.6 Å. By carefully examining the MD trajectories, it was speculated that the movement of the tB region directly resulted in the global movement of the Asp binding site and the local change of the AK structure. The structural change did make the *d*_Pi–O_ value larger and ATP move away from Asp. Based on the above ATP movement analysis, the possibility of the catalytic reaction was further reduced. The change of the local structure may greatly affect the catalytic efficiency of kinase. Fig. 4 gives the conformational differences of the residues of the Asp binding sites (the residues in direct contact with Asp) in the absence and presence of inhibitor lysine. As seen in Fig. 4a, when Lys is not bound to the inhibitor site, there is no conformational change that may determine the catalytic reaction. The residues around Asp play a pivotal role in maintaining the substrate binding with AK. Many amino acid residues forming H-bonds with Asp are very important to stabilize the phosphorylation, which are Ser59, Thr65, Glu92, Arg169, Gly171, and Ser172. The H-bonds between Glu92 and Asp could be regarded as the ionic bond formed by the side chain carboxyl group of Glu with the main chain amino group of Asp. However, the presence of Lys has influence on the interactions between Asp and its binding site residues. First, conformational translation occurred in the Arg169–Ser172 region. Secondly, many hydrogen bonds are destroyed, such as the H-bonds between Asp and the residues Thr65 and Gly171, and the H-bond between Ser59 and the main-chain amino group of Asp (Fig. 4b). The ionic bond between Glu92 and Asp is also absent. It was believed that the changes of these interactions would seriously damage the catalytic efficiency of kinase.

#### 2.3.4. Effect of mutations on resistance to feedback

**Fig. 5:**
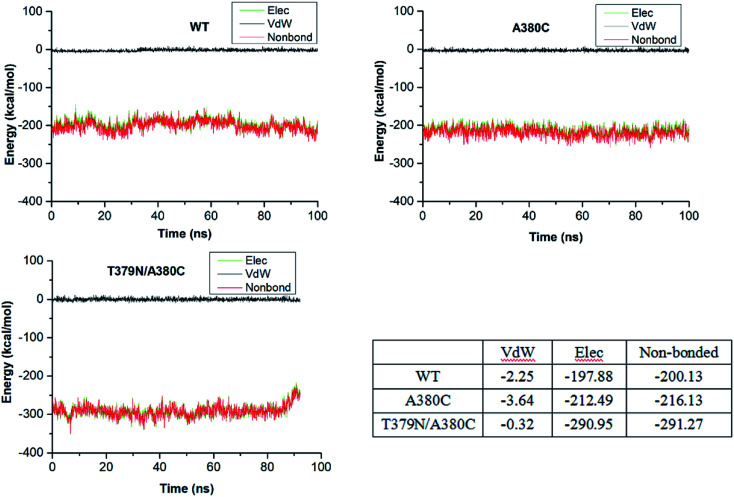
The interaction energy between the substrate Asp and AK protein.

Next, we would compare the total conformational change of the mutations A380C and T379N/A380C with WT. As shown in Fig. S4(A),† the superposition of the whole enzymes (WT, A380C, and T379N/A380C) is very good. Accordingly, it was supposed that the allosteric regulatory mechanism of Lys in the mutations was very similar to that of WT. Moreover, the conformation shift observed in WT + Lys does not appear in the mutant system. That is to say, mutations open the “switch” of allosteric regulatory, and resist the effect of inhibition because they can prevent conformational transformation of AK, thereby promoting the catalytic reaction although the inhibitor binds to the corresponding active site. Interestingly, it can be observed from Fig. S4(B)† that the two key residues R203 and D193 of T379N/A380C are closer to ATP than those of A380C. The result implied the enhanced enzymatic activity of the double-mutant. Additionally, the catalytic residue G168 in A380C and T379N/A380C forms a H-bond with Asp. The additional H-bond between the residue G168 and Asp might be more conducive to enzyme catalyzation, so the enzyme activity of the mutants is improved. In order to understand the enzyme catalyzation in depth, the interaction energy between the substrate Asp and AK was calculated (Fig. 5). It can be observed that their van der Waals are close to zero, and the interaction energy is electrostatic interaction energy mainly. The electrostatic interaction energy of WT, A380C, and T379N/A380C is 197.88 kcal mol^−1^, −212.49 kcal mol^−1^, and −290.95 kcal mol^−1^, respectively. For a point mutation, the relative interaction energy between the wild type and the mutant A380C is 14.61 kcal mol^−1^, but that of T379N/A380C is 93.07 kcal mol^−1^. The calculated results indicated that the interaction between substrate Asp and AK of the double mutation is much stronger than that of the single mutation. The stronger interaction can reflect enhanced affinity, which is consistent with the experimental MST data. The *K*_d_ values for WT, A380C + Lys, and T379N/A380C + Lys are 655.08, 447.69, and 99.64 mM, respectively. The *K*_d_ value of the double mutation becomes much lower, indicating its stronger affinity between Asp and AK. Also, the double-mutant shows higher the enzyme activity (22.79-fold relative to the wild type) than the single mutant (12.35-fold). At the same time, the residues around the substrates ATP or Asp are being mutated on the basis of T379N/A380C for enhancing enzyme activity. Also, the enzyme activity improvement of multipoint mutations as well as the mechanism of AK catalyzation are being in depth studied in our team.

## 3. Materials and methods

### 3.1. Experimental materials

Recombinant plasmid pET-28a–AK from the *Escherichia coli* was preserved by our laboratory. The plasmid extraction kit, protein electrophoresis marker, nucleic acid electrophoresis marker, and DpnI digestive enzyme were purchased from the TaKaRa Company (Japan). His Trap HP 5 mL was bought from General Electric Company (USA), and His-Tag (27 × 10^8^) Mouse mAb (HRP conjugate) (art. no. 9991S) from Cell Signaling Technology (USA). Monolith™ Protein Labeling Kit RED-NHS and Capillaries for Monolith NT115 Standard Capillary were bought from NanoTemper Technology Co., Ltd. (Beijing, China).

### 3.2. Construction of mutant strains

To construct mutants of *Cp*AK, the sites T379N and A380C were subjected to site-directed mutagenesis. According to the design principle of site-directed mutagenesis, the mutant primers were as follows: A380 – 5′GCTTCCATGAACTCNNNGGTAACACCTGG3′, 5′CCCAGGTGTTACCNNNGAGTTCATGGAAG3′; T379N/A380C – 5′CAGAGCTTCCATGAACTCACANNNAACACCTGGGTGAG3′, 5′CAGTCTCACCCAGGTGTTNNNTGTGAGTTCATGGAAGC3′. PCRs were performed to amplify the target fragment sequences employing the recombinant plasmid pET-28a–AK as the template. The resulting plasmid were then digested by DpnI enzyme under the conditions: DpnI enzyme 0.3 μL, buffer 2 μL, PCR product 2 μL, and at 37 °C for 2 h. The PCR products were transformed into *E. coli* BL21 cells by the method of heat shock, which contained an ice bath for 5 min, hot compress at 42 °C for 90 s, and an ice bath for 2 min again, adding 900 μL Luria–Bertani (LB) solid medium without resistance. After the cells were incubated at 37 °C, 160–170 rpm for 90 min, centrifuged at 8000 rpm for 2 min, and finally 800 μL supernatant were discarded.

Monoclonal strains expressing mutant AK with high-enzyme activity were achieved by high-throughput technique.^16^ The high-throughput method was as follows: the mutant strains were collected as much as possible, cultivated in the 96-well plate (200 μL LB culture medium) at 37 °C, 180 rpm for 12 h, and then 20 μL strains were transferred to a new 96-well plate for inducing 4.5 h at 37 °C, 200 rpm. After 1 mM isopropyl-β-d-thiogalactopyranoside (IPTG) was added into the strains which were induced at 30 °C, 130 rpm for 8 to 9 h. The cells were gathered by centrifugation at 3500 rpm for 45 min, and then frozen at −80 °C for 0.5–1 h and thawed at 30 °C for 0.5–1 h. The crude enzymes could be obtained and their enzyme activity were determined in the absorbance at 600 nm by high throughput screening.

The strains with high-enzyme activity were amplified with the cloned primers (5′GGAATTCCATATGGCCCTGGTCGTACAGAA3′, 5′GGAATTCTTAGCGTCCGGTGCCTGCAT3′) and PCR amplification of bacterial liquid was carried out. After the products were verified by 1% agarose gel electrophoresis. The *Cp*AK gene replacement mutant strains were further verified by DNA sequencing and for subsequent use.

### 3.3. Purification and validation of AK

The bacteria solution of successful sequencing was induced by 1 mM isopropyl-β-d-thiogalactoside (IPTG) overnight at 23 °C. The cells were subsequently collected after centrifugation at 4 °C, 8000 rpm for 10 min, and suspended in 10 mL PBS (pH 7.4). The suspension was centrifuged again for 10 min after ultrasonic crushing, and purified with ÄKTA. The conditions were as follows: (1) equilibration: PBS of 10 CV (column volume); flow rate, 5 mL min^−1^; pressure, 0.4 MPa; (2) sample application: sample volume, 6 CV; flow rate, 2 mL min^−1^, pressure, 0.4 MPa; (3) equilibration: PBS of 25 CV; (4) elution: elution was performed by a linear gradient of buffer B (500 mM imidazole) and buffer A (20 mM imidazole). The concentration of buffer B was from 0 to 35% during 20 CV and then eluted by 100% with 25 CV. The solution was 4 mL per tube, and the purified liquid was verified by SDS-PAGE and western blot.

### 3.4. Kinetic assays

The reaction system (1 mL) contained at the final concentration: 1.6 mM MgSO_4_, 800 mM KCl, 0.5–16 mM l-Asp, 10.4 mM ATP (dissoluted by 25 mM Tris–HCl), 100 mM Tris–HCl buffer solution (pH 8.0), 10 mM β-mercaptoethanol, and 800 mM NH_4_OH. The water was instead of l-Asp as blank control. The reaction was generated by adding 50 μL purified enzyme at 28 °C for 30 min, and stopped by adding FeCl_3_ solution (three parallel, below the same) and the absorbance were determined at 540 nm. The nonlinear curve was fitted by the Hill equation (*V* = *V*_max_ (*S*^*n*^)/(*K*^*n*^ + *S*^*n*^)) using Origin 8.5 software. In the equation, the *n* value represents the Hill coefficient; the *K* value is equal to the substrate concentration at half the maximum rate of enzymatic reaction; *V*_max_ and *V* indicate the maximum velocity and reaction rate, respectively; *S* means the substrate concentration.

### 3.5. Determination of enzymology properties

The reaction was performed through adding 50 μL purified enzyme at different temperature gradients (15, 20, 25, 26, 28, 30, 35, 40, 45, and 50 °C) and different pH gradients (6.0, 6.5, 7.0, 7.5, 8.0, 8.5, 9.0, 9.5, and 10.0) for 30 min, severally. It was terminated by adding FeCl_3_ and measured absorbance at 540 nm. l-Asp was instead of water as negative control. The highest enzyme activity was defined as 100%.

The reaction was performed through adding 50 μL purified enzyme at the optimum temperature and pH. The initial enzyme activity was defined as 100% and then measured every one hour in total 10 times. The half-life period was defined by the half of the original enzyme activity.

The reaction was performed through adding 20 μL purified enzyme and 20 μL substrate inhibitors (Lys, Thr, Met, Lys + Thr, Lys + Met, Thr + Met, and Lys + Thr + Met) at 0.2, 1, 5, and 10 mM. The reaction was finished by adding volume FeCl_3_ after 30 min and the absorbance was measured at 540 nm. Water was instead of the substrate inhibitor as blank control. The enzyme activity of blank control was defined as 100%.

### 3.6. Microscale thermophoresis (MST) analysis

The purified AK concentration was adjusted to 2–20 μM with labeled buffer. The solid fluorescent dye (NT-647-NHS) was appended with 100% DMSO (30 μL) and dissolved thoroughly after vortex vibration. Then the dyes were adjusted to 2–3 folds of the protein concentration with the marker buffer. Protein and the solid fluorescent dye were mingled in equal proportion for dark reaction 30 min, and then purified according to the purification kit operation. The unlabeled substrate Asp and inhibitor Lys were diluted in the enzymatic activity measurement system, and the final concentration ranged from 0.5 M to 15 M and from 0.1 M to 3 M, respectively, which they were incubated with the labeled protein (40 nM) at room temperature for 10 min. Then the samples were loaded into the Standard Capillaries from Nano Temper Technologies. Thermophoresis was measured 30 min after incubation at 28 °C on Monolith NT.115.^22^ The dissociation constant (*K*_d_) values were fitted by means of the MO. Affinity analysis software.

### 3.7. Molecular dynamics simulations

In our previous studies,^17,18^ the homology model for *Cp*AK was obtained. Here we directly used the homology model, including the ligands (Asp and ATP) and inhibitor (Lys). The mutants of AK were generated by Discovery Studio (DS).^23^ All of the missing atoms in the complex are automatically added by the VMD psfgen plugin.^24^ Then more TIP3P molecules^25^ extending up to 10 Å from the solute in each direction were added by the solvate plugin in VMD, forming a rectangular solvated system. The salt concentration of the system was set to 154 mM by the addition of Na^+^ and Cl^−^ counterions.

All the molecular dynamics (MD) were performed by the package NAMD 2.12.^26^ Topology and force field parameters were assigned from the CHARMM36 parameter set.^27^ The van der Waals and short-range electrostatic interactions were gradually turned off at a distance range between 9 and 10 Å. The long-range electrostatic interactions were described by the particle mesh Ewald (PME).^28^ Every simulation system was first energy minimized with 20 000 steps of conjugate gradients, by restraining the positions of the protein backbone and ligand atoms with a force constant of 1 kcal mol^−1^ Å^−2^. After that, at the same restraint, the temperature of the system was, using Langevin thermostat,^29^ gradually increased from 100 to 300 K at an increment rate of 10 ps K^−1^ within the simulation time of 2 ns. Next, 1 ns MD simulation was carried out to ensure the stability of the system's temperature in the canonical (NVT) ensemble, and then 2.5 ns NPT MD simulations were performed in which the pressure of the system was coupled to a reference pressure of 1 bar with the modified Nosé–Hoover–Langevin piston method.^30^ In the process, the restraint potential function for the system was gradually turn off by progressively decreasing the scaling factor to 0. Finally, at least 110 ns free NPT MD simulations were conducted. The MD trajectories were analyzed using VMD. Root-mean-square deviation (rmsd) of the Cα atoms was calculated.

## 4. Conclusions

*Corynebacterium pekinense* AK was successfully modified and two mutants A380C and T379N/A380C with high enzyme activity (12.35-fold and 22.79-fold as compared to *Cp*AK, respectively) were constructed. The structural and biochemical analyses such as enzyme activity are used by both experimental and theoretical methods. The microscale thermophoresis results showed that the mutations in sites T379 and 380 can resist the feedback inhibition of Lys. Moreover, the molecular dynamics simulation has given a reasonable explanation of the inhibitor mechanism of inhibitor Lys as well as the feedback inhibition-resistant of mutations. The binding of Lys with WT resulted in conformational changes of *Cp*AK and a larger distance between the phosphorus atom of ATP and the oxygen atom of Asp (*d*_Pi–O_ = 4–5 Å), and thus, was detrimental for the catalytic reaction. However, mutations can present the conformational transformation and some key residues such as G168, R203, and D193 play an important role in maintaining the substrates binding with *Cp*AK and further enhance the enzyme activity. The *K*_d_ value of Asp and AK experimented by MST for WT, A380C + Lys, and T379N/A380C + Lys is decreased and the affinity of substrate Asp and AK is enhanced, which is in good agreement with the increased electrostatic interaction energy. The present work is useful for the further study of phosphorylation mechanism of aspartate kinase, and it provides the rational engineering of the biosynthesis pathway of amino acid in the production of Asp-derived amino acids.

## Conflicts of interest

The authors declare no competing financial interests.

## Supplementary Material

RA-011-D0RA09153G-s001
