# Supplementary material for: Mechanism of the feedback-inhibition resistance in aspartate kinase of Corynebacterium pekinense: from experiment to MD simulations
Source: RSC Adv. 2020 Dec 22;11(1):30–8. doi: 10.1039/d0ra09153g (PMC8690038; doi:10.1039/d0ra09153g)
Supplement: RA-011-D0RA09153G-s001 [file RA-011-D0RA09153G-s001.pdf]

Electronic Supplementary Material (ESI) for RSC Advances.
This journal is © The Royal Society of Chemistry 2020

Electronic Supporting Information

Mechanism of the Feedback-Inhibition Resistance in Aspartate Kinase of *Corynebacterium pekinense*: from Experiment to MD Simulations

Xiaoting Liu,‡[ab] Caijing Han,‡[abc] Li Fang,[ab] Zhanqing Fan,[ab] Yanan Wang,[ab] Xin Gao,[ab] Junhua Shi,[ab] and Weihong Min*[ab]

[a] College of Food Science and Engineering, National Engineering Laboratory of Wheat and Corn Deep Processing, Jilin Agricultural University, Changchun 130118, Jilin, People's Republic of China.

[b] National Engineering Laboratory of Wheat and Corn Deep Processing, Changchun 130118, Jilin, China.

[c] School of Public Health Weifang Medical University, Weifang 261042, Shandong, China.

*To whom correspondence should be addressed. E-mail: minwh2000@jlau.edu.cn. Tel: +86-139-4491-9697. Fax: +86-431-8451-7235.

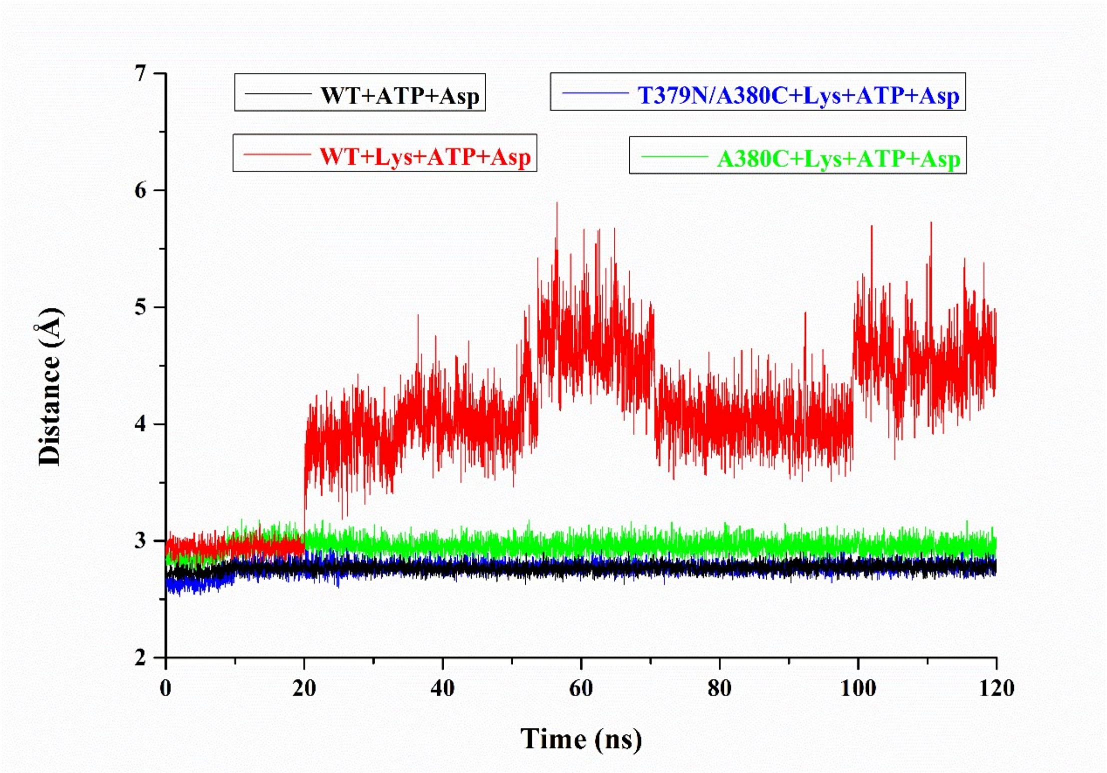

Fig. S1 The $d_{Pi\text{-}O}$ (the distance between the phosphorus atom of ATP and the oxygen atom of Asp) changes with the simulation time.

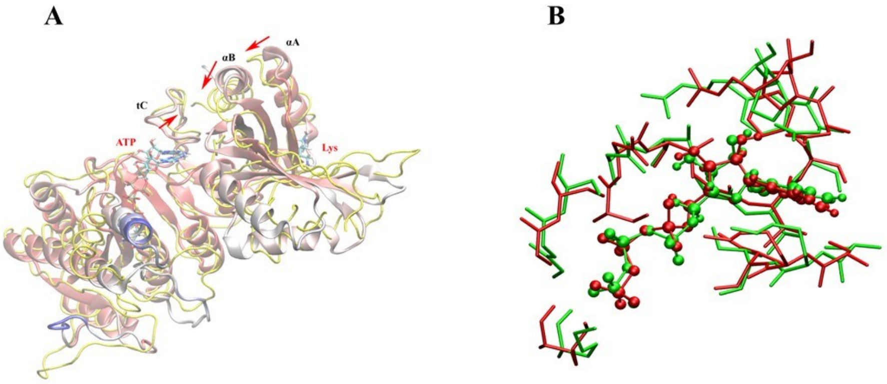

Fig. S2 Effect of inhibitor Lys on ATP Binding for WT. (A) Effect path of inhibitor Lys on ATP binding, the cartoon structure in yellow shows the conformation with inhibitor with Lys; (B) Alignment of binding site amino acids of ATP in the absence (green) and presence (red) of Lys, ATP is represented as CPK model.

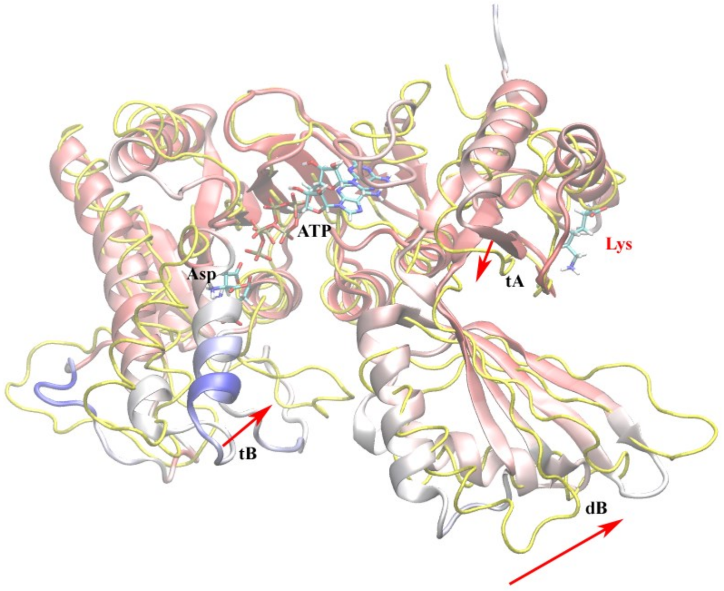

Fig. S3 Conformational changes between WT and WT+Lys. The WT+Lys system was represented as yellow, while ATP, ASP, and inhibitor Lys were represented as CPK model.

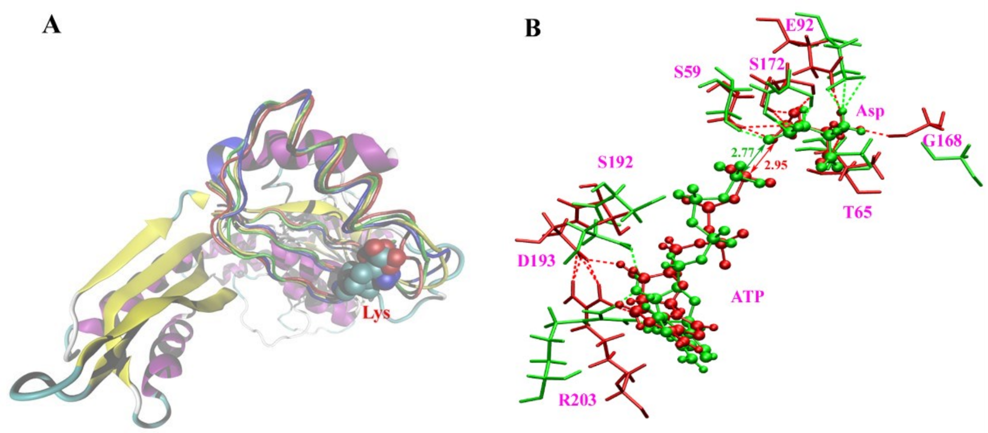

Fig. S4 Allosteric regulatory of WT and mutants. (A) the different conformation of Lys binding site for mutant A380C (blue) and T379N/A380C (yellow) as well as WT (red) without Lys systems, Lys is shown as VDW. (B) The change of residues around the substrate Asp. The sticks from A380C and T379N/A380C are colored by red and green, respectively.
